# A survey of *Anopheles* species composition and insecticide resistance on the island of Bubaque, Bijagos Archipelago, Guinea-Bissau

**DOI:** 10.1186/s12936-020-3115-1

**Published:** 2020-01-15

**Authors:** Thomas Ant, Erin Foley, Scott Tytheridge, Colin Johnston, Adriana Goncalves, Sainey Ceesay, Mamadou Ousmane Ndiath, Muna Affara, Julien Martinez, Elizabeth Pretorius, Chris Grundy, Amabelia Rodrigues, Paulo Djata, Umberto d’Alessandro, Robin Bailey, David Mabey, Anna Last, James G. Logan

**Affiliations:** 1 Department of Disease Control, Faculty of Infectious and Tropical Diseases, London School of Hygiene and Tropical Medicine, Keppel Street, London, WC1E 7HT UK; 2 Clinical Research Department, Faculty of Infectious and Tropical Diseases, London School of Hygiene and Tropical Medicine, Keppel Street, London, WC1E 7HT UK; 3 Disease Control & Elimination Theme, Medical Research Council Unit, London School of Hygiene and Tropical Medicine, Fajara, The Gambia; 4 Department of Infectious Disease Epidemiology, Faculty of Epidemiology and Population Health, London School of Hygiene and Tropical Medicine, Keppel Street, London, WC1E 7HT UK; 5 Bandim Health Project, INDEPTH Network, Bissau, Guinea-Bissau; 6 Ministério da saude chez Ministério da saude de Guinea-Bissau, Bissau, Guinea-Bissau; 7 MRC The Gambia at the London School of Hygiene & Tropical Medicine, Fajara, Gambia; 8 ARCTEC, Chariot Innovations Ltd, London School of Hygiene and Tropical Medicine, Keppel Street, London, WC1E 7HT UK

**Keywords:** Malaria, Guinea-Bissau, Bijagos islands, vector survey, Vector control, Anopheles gambiae, Insecticide resistance

## Abstract

**Background:**

Bubaque is the most populous island of the Bijagos archipelago, a group of malaria-endemic islands situated off the coast of Guinea-Bissau, West Africa. Malaria vector control on Bubaque relies almost exclusively on the use of long-lasting insecticidal nets (LLINs). However, there is little information on local vector bionomics and insecticide resistance.

**Methods:**

A survey of mosquito species composition was performed at the onset of the wet season (June/July) and the beginning of the dry season (November/December). Sampling was performed using indoor adult light-traps and larval dipping. *Anopheles* mosquitoes were identified to species level and assessed for *kdr* allele frequency by TaqMan PCR. Females were analysed for sporozoite positivity by CSP-ELISA. Resistance to permethrin and α-cypermethrin was measured using the CDC-bottle bioassay incorporating the synergist piperonyl-butoxide.

**Results:**

Several *Anopheles* species were found on the island, all belonging to the *Anopheles gambiae* sensu lato (s.l.) complex, including *An. gambiae* sensu stricto, *Anopheles coluzzii*, *Anopheles melas*, and *An. gambiae*/*An. coluzzii* hybrids. Endophagic *Anopheles* species composition and abundance showed strong seasonal variation, with a majority of *An. gambiae* (50% of adults collected) caught in June/July, while *An. melas* was dominant in November/December (83.9% of adults collected). *Anopheles gambiae* had the highest sporozoite rate in both seasons, with infection rates of 13.9% and 20% in June/July and November/December, respectively. Moderate frequencies of the West African *kdr* allele were found in *An. gambiae* (36%), *An. coluzzii* (35%), *An. gambiae*/*An. coluzzii* hybrids (42%). Bioassays suggest moderate resistance to α-cypermethrin, but full susceptibility to permethrin.

**Conclusions:**

The island of Bubaque maintained an *An. gambiae* s.l. population in both June/July and November/December. *Anopheles gambiae* was the primary vector at the onset of the wet season, while *An. melas* is likely to be responsible for most dry season transmission. There was moderate *kdr* allele frequency and synergist assays suggest likely metabolic resistance, which could reduce the efficacy of LLINs. Future control of malaria on the islands should consider the seasonal shift in mosquito species, and should employ continuous monitoring for insecticide resistance.

## Background

Bubaque is the most populated island and the main commercial centre of the Bijagos archipelago, a group of 88 remote islands and islets situated off the coast of Guinea-Bissau, West Africa. A majority of the approximately 30,000 Bijagos islanders maintain a lifestyle supported by subsistence agriculture, with the port on Bubaque acting as the major hub for the import and export of goods, and the movement of people between the islands and the mainland. Bubaque consists primarily of dense forest and areas of cultivated agricultural land surrounded by stretches of beach and mangrove swamp, although the majority of the island’s approximately 11,000 inhabitants are concentrated in a semi-urbanized area built around the port. The climate is tropical, with a heavy wet season between June and November, and a dry season from December to May.

Malaria is a leading public health concern in Guinea-Bissau, with an estimated parasitological prevalence ranging from 3 to 30% [1, 2], and a peak in transmission towards the end of the wet season (October–November) [3]. Although several entomological surveys have been completed in and around the capital city of Bissau, there is limited knowledge of vector species composition, sporozoite rate, and insecticide resistance on the Bijagos islands. To our knowledge there has been only one previous mosquito survey at a single site on Bubaque that reported a mixture of primarily *Anopheles gambiae* sensu stricto (s.s.) (hereafter *Anopheles gambiae*)*, Anopheles coluzzii*, *An. gambiae*/*An. coluzzii* hybrids, and a low number of the salt-tolerant species *Anopheles melas* [4]. In mainland Guinea-Bissau, *An. gambiae*, *An. coluzzii,* and their hybrids are the dominant malaria vectors [5], with *An. gambiae* having the highest sporozoite rate [5]. *Anopheles melas* often breeds at high densities in coastal regions of West Africa [6, 7], and sporozoite-positive females have been reported in coastal areas of Guinea-Bissau [5].

Malaria vector control on Bubaque currently relies on the use of long-lasting insecticidal nets (LLINs). Government-led LLIN distributions have been implemented on a 3-year cycle since 2011, and household surveys suggest high uptake (> 90%, Anna Last, unpublished data). Although LLIN-based vector control has contributed to impressive reductions in malaria prevalence across Africa, selection for pyrethroid resistance has the potential to slow and perhaps even reverse this progress [8]. As a result, the WHO recommends regular insecticide resistance monitoring in areas with widespread LLIN use.

The island ecology of the Bijagos archipelago creates a potential for differences in malaria vector composition and seasonality compared to mainland Guinea-Bissau. To design/maintain an appropriate malaria control programme for the islands, a comprehensive understanding of the malaria vectors and their dynamics is needed. An entomological investigation on the Bijagos archipelago is presented here, describing the major malaria vectors on the island of Bubaque, their seasonal variation and sporozoite rates, and a characterisation of resistance to pyrethroid insecticides.

## Methods

### Study site and sampling locations

**Fig. 1:**
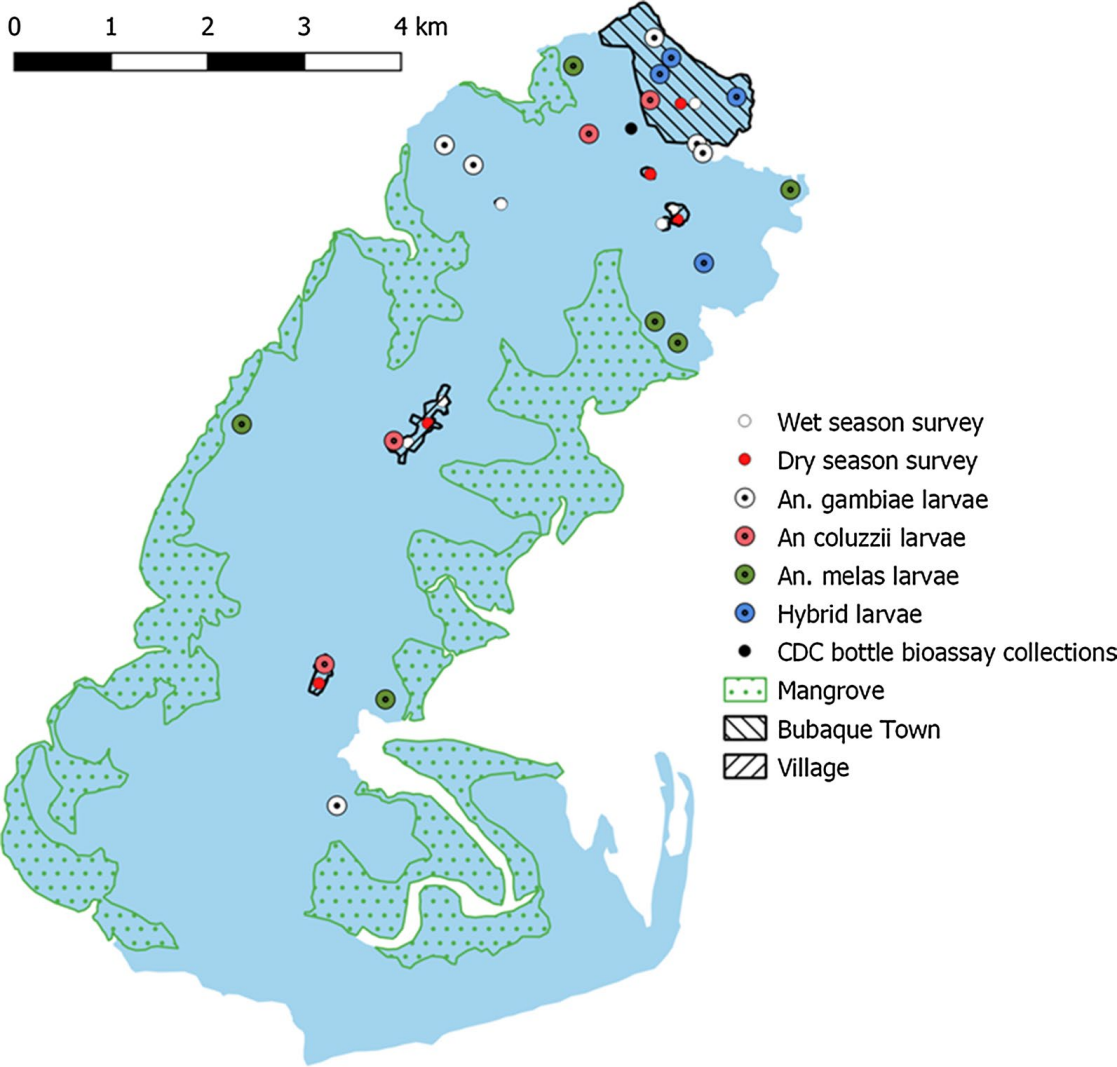
The island of Bubaque in the Bijagos archipelago of Guinea-Bissau. Map shows the locations of adult and larval sampling

The island of Bubaque is approximately 35 km from the mainland of Guinea-Bissau and comprises an area of around 85 km^2^. It contains several habitat types, including a semi-urban town, rural villages, dense forest, savannah, floodplain, extensive mangroves, and temporary and permanent agricultural areas. Mosquito adult sampling was performed in houses located in Bubaque town and the villages of Anbanha, Ancadona, Bijante and Brus (Fig. 1). A household census survey, carried out at the beginning of this pilot study, revealed that there are typically 2–3 households per building. Mosquito sampling was performed over the months of June/July 2017 (the onset of the rainy season) and then again over the months of November/December 2017 (the end of the rainy/onset of the dry season).

### Adult trapping

Study villages were selected at random using probability proportional to size sampling. Before sampling, the households in a village were surveyed and numbered. A random sample of households was chosen to meet the sample size requirements. On average, 15 households were sampled per village. Indoor biting adult mosquitoes were collected from inside the occupied bedrooms of local houses using CDC miniature light traps (John W. Hock Company). Traps were hung with a bulb height of approximately 150 cm and 50 cm from the foot of an occupied bed. Traps were active for the duration of the night. An electronic questionnaire was completed upon trap collection, in which it was established from the occupants and visual inspection whether the trap had been running all night.

### Larval sampling

Random GPS coordinates were generated on Bubaque using the “*create random points*” and “*trace*” commands in ArcGIS. A 20 × 20 m quadrant was established at a given GPS point and potential mosquito larval habitats were identified by walking from one end of the quadrant to the other. A standard 350 ml larval dipper and a large 30 ml pipette were used for collection of larvae. Each sampling site received several sampling replicates dependant on the size of the water body. Larval habitats were grouped into one of four size categories: 0.01–1 m, 1.01–10 m, 10.01–100 m and > 100 m and this dictated the number of dips per site—3, 10, 15 or 50 dips [9]. Larval habitats were characterized according to location, type, and size. When present, all larvae and pupae were collected in vials with a larger amount of breeding water also taken for later rearing. Rearing was performed in standard larval trays using water collected from the breeding site when possible.

### Identification and sample storage

Adult mosquitoes caught by CDC light trap were immediately sacrificed by exposure to chloroform and morphologically identified using the keys of Gillies and Coetzee [10] and Gillies and De Meillon [11]. Adult mosquitoes emerging from sampled larvae were either sacrificed by chloroform or were kept alive for use in downstream bioassays. Sacrificed anopheline adults were stored by desiccation in 1.5 ml microcentrifuge tubes with cotton wool and silica gel beads and placed in a cool and dark place until molecular analysis.

### DNA extraction

DNA was extracted from mosquitoes using the automated QIA cube Extractor robot (Qiagen) following the manufacturer instructions. The mosquito parts (head and thorax) were lysed by incubation in a mixture of proteinase K and buffer ATL solution overnight at 56 °C on a shaking incubator. The digest elutes were transferred into lysis 96-well plate and placed in the extraction robot. The elutes were finally transferred to capture plates, washed and DNA eluted into 80 μl and stored at − 20 °C.

### *kdr* genotyping

Knock down resistance assay was performed in 10 µl reaction volume containing 2× TaqMan mix, 1× primer/probe mix and 1 µl template DNA, using TaqMan assay for insecticide resistance markers Vgsc 1014F (Kdr west) and Vgsc 1014S (Kdr east) mutations in the Voltage gated sodium channel gene that confer resistance to DDT/pyrethroids in *An. gambiae* complex.

*kdr east* was run at initial denaturation of 95 °C for 10 min followed by 45 cycles of 92 °C for 10 s and 60 °C for 1 min by using the primers described by Ranson et al. [12], while *kdr west* was run at an initial denaturation of 95 °C for 10 min followed by 45 cycles of 95 °C for 10 s and 60 °C for 45 s according to the protocol described by Martinez-Torres et al. [13], using the CFX 96 Bio-Rad real time PCR Machine. The VIC and FAM fluorescence was captured at the end of each cycle and genotypes called from endpoint fluorescence using the Bio-Rad CFX 96 software.

### *Anopheles gambiae* sensu lato species identification

Scott’s PCR was carried out by using the following primers (UN (F) [GTGTGCCCCTTCCTCGATGT], AR (R) [AAGTGTCCTTCTCCATCCTA], GA (R) [CTGGTTTGGTCGGCACGTTT], ME (R) [TGACCAACCCACTCCCTTGA]) [14] to detect *An. gambiae*, *An. arabiensis* and *An. melas*. Hha I was used to digest the PCR product [15] to further distinguish between *An. gambiae* and *An. coluzzii* using the Bio-Rad T100 PCR Machine.

Amplified products were run using the QIAxcel capillary electrophoresis system (Qiagen), using the screening cartridge, 15–1000 bp alignment marker and 50–800 bp reference marker. The results were exported and double scored manually by visualizing gel image and the discrepancies sorted out by a third independent scorer.

### CSP ELISA

Mosquito’s head and thorax were placed in a 1.5 ml micro centrifuge tube, then ground in BB NP40 solution. Circumsporozoite protein (CSP) Elisa was performed on the mosquito triturate and plates read at 405 nm to detect the presence of CSP antigen [16].

### CDC bottle bioassays

*Anopheles* larvae were collected from an inland temporary breeding site close to the semi-rural Bubaque village in mid-June 2018 (Fig. 1) and were reared to pupation in a field insectary. Upon eclosion, adults were maintained in a 35 × 35 × 35 cm insect rearing cage with ad libitum access to a 10% sucrose solution. CDC bottle bioassays were conducted with adult females aged between 2 and 5 days [17]. Susceptibility to both permethrin (analytical-grade, Sigma Aldrich, St. Louis, Missouri) and alpha-cypermethrin (analytical-grade, Sigma Aldrich, St Louis, Missouri) was measured at discriminating doses of 21.5 and 12.5 µg/bottle, respectively, using a discriminating time of 30 min. The bottle bioassay was repeated for alpha-cypermethrin with the addition of a 1-h pre-exposure to the synergist piperonyl-butoxide (PBO) at a dose of 400 µg/bottle (analytical-grade, Sigma Aldrich, St. Louis, Missouri). PBO-only exposure controls were also performed. The mortality in the control group (unexposed to insecticide) was consistently less than 5%.

## Results

### Anopheline species composition and abundance

A total of 1869 adult mosquitoes (853 anopheline and 1016 culicine), were collected by CDC lights traps. 721 female anopheline mosquitoes were collected in June/July (the onset of the wet season) while 126 were collected in November/December (the end of the wet/onset of the dry season). All *Anopheles* specimens were morphologically identified as *An. gambiae* sensu lato (s.l.). A sub-set of randomly selected samples (214 in total) from the June/July collections and all samples from November/December were identified to species level by PCR.

In June–July, *An. gambiae* was the predominant species (50%), followed by *An. coluzzii* (12.6%), *An. gambiae*/*An. coluzzii* hybrids (27.5%), and *Anopheles melas* (9.8%). In November/December, *An. melas* was the predominant species (83.9%), with smaller proportions of *An. gambiae* (4.2%), *An. coluzzii* (2.5%) and *An. gambiae*/*An. coluzzii* hybrids (9.3%) (Table 1). The median number of female *An. gambiae* s.l. caught in a single household trap per night was 8 in June/July, and one in November/December (Additional file 1: Figure S1).

**Table 1:** Numbers of *Anopheles gambiae* s.l. species caught by CDC light traps placed

Collection months	*Anopheles* species				Total
	*An. gambiae*	*An. coluzzii*	*An. gamb*/*col* hyb	*An. melas*	
June/July	107 (50%)	27 (12.6%)	59 (27.5%)	21 (9.8%)	214
November/December	5 (4.2%)	3 (2.5%)	11 (9.3%)	99 (83.9%)	118

Eight November/December samples did not successfully PCR amplify

### Sporozoite rates

Over June/July, CSP positivity rates were 13.9% for *An. gambiae*, 7.4% for *An. coluzzii*, 5.1% for *An. gambiae*/*An. coluzzii* hybrids, and 4.8% for *An. melas* (Table 2). This compares with 20% for *An. gambiae*, 9.1% for *An. gambiae*/*An. coluzzii* hybrids, and 6.1% for *An. melas* over November/December.

**Table 2:** Numbers and percentages of sporozoite-positive *Anopheles gambiae* s.l. species, as determined by CSP antigen ELISA

Collection period	June/July			November/December		
	Total positive	Total negative	Infection rate (%)	Total positive	Total negative	Infection rate (%)
*An. gambiae*	15	93	13.9	1	4	20
*An. coluzzii*	2	25	7.4	0	3	0
*An. gamb*/*col* hybrid	3	56	5.1	1	10	9.1
*An. melas*	1	20	4.8	6	93	6.1

### Larval collections

88 potential mosquito breeding sites were identified and assessed through larval dipping, of which 16 (18.2%) were positive for *Anopheles* larvae. A total of 83 *Anopheles* larvae were reared to adulthood, morphologically identified as *An. gambiae* s.l., and subsequently identified to species level by PCR. No additional *Anopheles* species were discovered over those previously identified in adult catches, with collections comprising *An. gambiae* (60.2%), *An. gambiae*/*An. coluzzii* hybrids (24.1%), *An. coluzzii* (7.3%) and *An. melas* (8.4%) larvae. The locations of the *Anopheles*-positive larval breeding sites are given in Fig. 1. While *An. gambiae*, *An. coluzzii* and *An. gambiae*/*An. coluzzii* hybrids were associated with urban and agricultural habitats, *An. melas* tended to be associated with coastal breeding sites. Culicine mosquitoes were also collected and identified as *Aedes aegypti*, *Culex rubinotus*, *Culex theileri*, and *Toxorhynchites* species. A complete list of species sampled including *Culicines* can be found in Additional file 2: Table S1.

### *kdr* frequencies

**Fig. 2:**
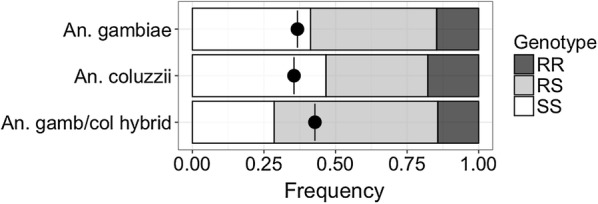
Genotype and allele frequencies of the West African (*kdr*-w) allele in *An. gambiae* s.l. Total individuals analysed (n) was 106 for *An. gambiae*, 27 for *An. coluzzii* and 59 for hybrid forms. *Anopheles melas* was also assessed for *kdr*-w but was negative in all samples (n = 33). Black dots with vertical lines indicate total *kdr*-w allele frequency

All adult *Anopheles* samples collected over June/July were tested for the presence of the ‘West African’ 1014F *kdr* allele (*kdr*-w) [13] and the ‘East African’ 1014S *kdr* allele (*kdr*-e) [12]. Frequencies of the *kdr*-w allele were 36% in *An. gambiae,* 35% in *An. coluzzii* and 42% in *An. gambiae*/*An. coluzzii* hybrids (Fig. 2). No *kdr*-w alleles were found in *An. melas.* All samples were negative for the *kdr*-e variant.

### Functional bioassays and resistance genotyping

**Fig. 3:**
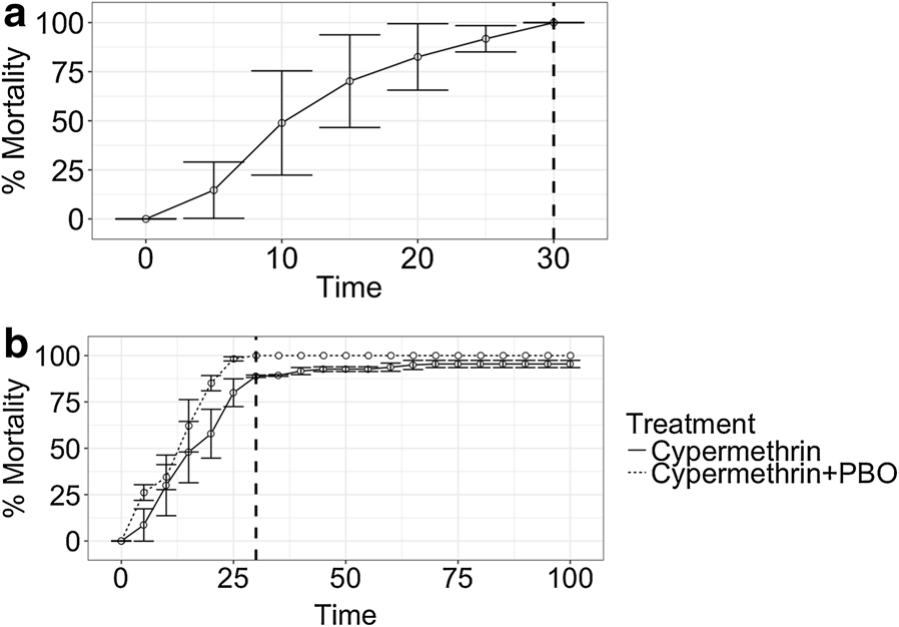
CDC bottle bioassay mortality curves for exposure to permethrin (**a**) and α-cypermethrin (**b**). Experiments with α-cypermethrin involved 2 separate treatments, one with α-cypermethrin-only, and one with an additional pre-exposure to piperonyl butoxide (PBO). Dotted vertical lines show diagnostic time when 100% mortality is expected in susceptible females

Larvae morphologically identified as *Anopheles* (and subsequently identified as adults as *An. gambiae* s.l.) were collected in breeding sites close to Bubaque village (Fig. 1). Age-matched adults reared from larval collections displayed full susceptibility to a discriminating dose of permethrin in CDC bottle bioassays, with 100% mortality of 204 *An. gambiae* s.l. females at the diagnostic time (Fig. 3a). However, incomplete mortality (172 out of 193 females, 89% ± 0.7%, 95% CI) was observed at the diagnostic time following exposure to α-cypermethrin, suggesting moderate levels of resistance to this pyrethroid [17] (Fig. 3b). To investigate potential mechanisms of resistance, a second batch of *An. gambiae* s.l. females was pre-exposed to the insecticide-synergist piperonyl butoxide (PBO), a compound that inhibits the action of mixed-function oxidases. The combination of PBO with subsequent exposure to α-cypermethrin resulted in 100% mortality (from 100 females), indicating the presence of metabolic resistance (Fig. 3b).

The species and *kdr*-w genotypes of all phenotypically resistant and a subset of susceptible mosquitoes used in the CDC bottle bioassays were identified by PCR (Table 3). There was a significant difference in *kdr*-w genotype frequencies among the resistant and susceptible *An. gambiae* (*kdr*-w frequency of 0.607 and 0.083 in phenotypically resistant and susceptible mosquitoes, respectively), and in *An. gambiae*/*coluzzii* hybrids (*kdr*-w frequency of 0.642 and 0.100 in resistant and susceptible mosquitoes, respectively). Carrying a copy of *kdr*-w allele was found to be strongly associated with α-cypermethrin resistance for both *An. gambiae* [odds ratio = 30 (3.56, 252.97 95% CI)] and *An. gambiae*/*coluzzii* hybrids [odds ratio = 24 (1.74, 330.8 95% CI)].

**Table 3:** *kdr*-w genotypes (R is the 1014F *kdr* resistant allele and S is the wild-type allele) of female *An. gambiae* s.l. mosquitoes phenotypically resistant and susceptible in CDC-bottle bioassays using α-cypermethrin

	Phenotype	Genotype			R allele frequency	Odds ratio (95% CI)
		SS	RS	RR		
An. gambiae	Resistant	2	7	5	0.607	30 (3.56, 252.97)
	Susceptible	10	4	0	0.083	
*An. gamb*/*col* hybrid	Resistant	1	3	3	0.642	24 (1.74, 330.8)
	Susceptible	8	2	0	0.100	

Numbers show total females of each genotype. Odds ratios (with 95% confidence intervals) were calculated to indicate the odds of an individual being resistant if it carried a *kdr*-w copy

## Discussion

The island of Bubaque displays strong seasonal variation in malaria vector species composition and abundance. During June/July, the onset of the rainy season, *An. gambiae* was the dominant endophilic species, although significant proportions of *An. coluzzii* and hybrid forms were also found. *Anopheles melas* adults constituted the smallest proportion of overall catches. The relative dominance of *An. gambiae* over *An. coluzzii* in this period indicates a reliance of the major portion of the wet season vector population on the availability of temporary, rain-dependent larval breeding sites (*An. coluzzii* tends to be associated with more permanent water bodies [18, 19]). In contrast, during November/December, *An. melas* was the dominant species (comprising > 85% of catches). This is likely a result of its capacity to maintain year-round populations by breeding in the island’s abundant littoral habitats and mangrove swamps (Bubaque is reasonably small, comprising a high ratio of coastal biome to inner-island area). Breeding of *An. gambiae* and *An. coluzzii* is limited to sites of low salinity, which are generally rare in the dry season. Catch rates per household suggest a greater absolute abundance of endophagic *An. gambiae* s.l. mosquitoes during the wet season (~ 8-fold higher per household catch rates compared with November/December), consistent with the increasing availability of temporary rain-dependent larval habitats supporting expanding mosquito populations.

A previous small-scale wet-season sample collected on Bubaque, reported a high proportion of *An. gambiae* mosquitoes (89.5%) (molecular forms not specified), with a lower proportion of *An. melas* (8.9%) [4, 20]. This is consistent with wet season surveys on mainland Guinea-Bissau, where *An. gambiae* and *An. coluzzii* were found to dominate [5, 21, 22]. Interestingly, Marsden and colleagues also reported catching a single *Anopheles arabiensis* adult on Bubaque (1 out of 67 *Anopheles* adults caught) [4, 20]. *Anopheles arabiensis* has also been found in the city of Bissau [23], and in the northern inland regions of Guinea-Bissau, where there is an abundance of its preferred dry shrubland and savannah habitats [24]. The lack of *An. arabiensis* in the present survey in either adult or larval collections likely reflects very low densities of this species on Bubaque, but may also result from a bias introduced by the indoor trapping method used in the large majority of mosquito collections; *An. arabiensis* tends to be less endophagic and endophilic than *An. gambiae* and *An. coluzzii* [25]. Larval collections closely reflected *Anopheles* species composition inferred from adult trapping, with a majority of *An. gambiae* and a smaller proportion of *An. gambiae*/*An. coluzzii* hybrids and *An. melas.* Several culicine species were also identified including the arbovirus vector *Aedes aegypti.*

Strong seasonal variation in abundance of *An. gambiae* s.l. species has been reported elsewhere in coastal regions of West Africa. A survey in The Gambia found reductions in overall *An. gambiae* s.l. numbers during the dry season, with catches dominated by *An. melas* [7]. Similarly, in a coastal region of southwest Nigeria the population of *An. gambiae* was highly seasonal and dependent on rainfall, while *An. melas* was able to maintain a relatively constant dry season population [6].

*Anopheles gambiae* and *An. coluzzii* exhibit strong reproductive isolation throughout the majority of their sympatric range, with rates of hybridization typically < 1% [26, 27]. However, higher rates of hybridization have been found in West Africa, particularly along the coastline of The Gambia [28] and Guinea-Bissau (where hybridization rates of up to 25% have been reported) [28–30]. A large proportion of hybrids were found on Bubaque, (hybrids accounting for > 27% of total catches, although 30.6% of *An. gambiae, An. coluzzii* and hybrid catches during June-July), with rates exceeding those commonly found on mainland Guinea-Bissau [5] and in The Gambia [28].

*Anopheles gambiae* had the highest *P. falciparum* sporozoite rate among all *An. gambiae* s.l. mosquitoes sampled, with 13.9% and 20% positivity in the dry and wet season, respectively, although the dry season sporozoite rates should be viewed with caution due to the very small sample size. The greater population density and sporozoite positivity of *An. gambiae* in the wet season suggests it is the primary wet season vector on Bubaque. Although *An. gambiae* also displayed the highest sporozoite rate of the *An. gambiae* s.l. species in the dry season, a far greater density of *An. melas* (with a 20-fold higher median per trap catch rate in this period) combined with a positive sporozoite rate of 6.1%, suggests that this species may be responsible for the majority of dry season transmission. The sporozoite rates found in the present survey are similar to those observed in a previous wet season survey on mainland Guinea-Bissau that included sampling on several of the islands of the Bijagos, where average sporozoite rates of 12.6% in *An. gambiae* and 11.1% in *An. melas* were recorded [5]. The higher sporozoite rates in *An. gambiae*, *An. gambiae*/*An. coluzzii* hybrids, and *An. melas*, at the onset of the dry season correlates with months of peak malaria prevalence. However, there was a lower average dry season sporozoite rate across the wider *An. gambiae* s.l., complex (9.7% in the wet season compared to 6.8% in the dry season) combined with ~ 8-fold lower indoor dry season catch rates. A tentative calculation of sporozoite-positive *An. gambiae* s.l. during both seasons, gives a median of 0.78 positive females per trap per night in the wet season, compared with 0.068 in the dry season.

Analysis of the wet-season samples from Bubaque revealed *kdr*-w allele frequencies of 36% in *An. gambiae,* 35% in *An. coluzzii* and 42% in *An. gambiae*/*An. coluzzii* hybrids. This contrasts with a survey in Bissau performed over a decade ago, where *kdr*-w allele frequencies of 7% and 0.8% were recorded in *An. gambiae* and *An. coluzzii*, respectively [31]. The sharp increase in *kdr*-w frequencies is consistent with a response to strong selection associated with the wide-spread distribution of LLINs in Guinea-Bissau since 2011.

Parallel comparisons between distributions of the *kdr* allele and pyrethroid resistance detected by in vivo assays are important for providing functional data on resistance, and may uncover additional mechanisms. Resistance can arise through a variety of processes, including metabolic resistance resulting from the amplification or upregulation of genes encoding detoxification enzymes. CDC-bottle bioassays were used in the present study to measure the resistance of field-caught *An. gambiae* s.l. females to discriminating doses of permethrin and α-cypermethrin. Although this represents a limited subset of insecticides used in LLINs, it represents two of the most commonly used pyrethroids in LLINs on Bubaque (Anna Last, unpublished data). Full susceptibility to permethrin and partial resistance to α-cypermethrin was found. This permethrin susceptibility contrasts with studies in the neighbouring Gambia, where marked resistance has previously been described, particularly in coastal areas [32]. FSusceptibility to α-cypermethrin was restored upon pre-exposure of mosquitoes to the insecticide synergist piperonyl butoxide (PBO), implicating mixed-function oxidases as an additional resistance mechanism. Mixed-function oxidase-based insecticide resistance is not uncommon among *An. gambiae* populations in West Africa, with biochemical assays and microarray studies implicating metabolic resistance in Benin [33], Nigeria [33], and Ghana [34]. Next generation LLINs (incorporating PBO) are currently being trialled in Africa in areas of high metabolic resistance, and have shown field efficacy [35], although, beneficial effects of PBO-nets may be restricted to areas with high levels of pyrethroid resistance [36].

## Conclusions

Endophilic *Anopheles* species composition and abundance on the island of Bubaque varied significantly by season, with a relatively large wet season population comprising principally *An. gambiae*, *An. coluzzii* and *An. gambiae*/*An. coluzzii* hybrids, and a smaller dry season population dominated by *An. melas.* The persistence of *An. melas* in the dry season, albeit at a low relative density, may be an important consideration for vector control planning. Information dissemination on LLIN use, for example, should stress the potential for malaria transmission and the benefits of LLIN use beyond the rainy season. Moderate frequencies of the West African *kdr*-mutation were found in both *An. gambiae* and *An. coluzzii* populations, and a low level of resistance to α-cypermethrin was detected through bioassay. Pre-exposure to PBO restored α-cypermethrin susceptibility, indicating the co-occurrence of both target-site and metabolic resistance mechanisms. The emergence of pyrethroid resistance is a serious concern on an island dependent on LLINs for malaria control. The results presented here suggest that resistance should be closely monitored by bioassay, and that the active insecticide compounds used in LLINs should be selected based on net-exposure assays with locally caught *An. gambiae* s.l. mosquitoes.

## Supplementary information

**Additional file 1: Figure S1.** Number of female *Anopheles* mosquitoes caught per CDC light trap per night during the June-July and November–December trapping.

**Additional file 2: Table S1.** Larval sampling site description and species found.

## Data Availability

All data presented in this manuscript are available from the corresponding author upon reasonable request.

## Abbreviations

LLIN: long-lasting insecticidal net
CSP: circumsporozoite protein
PBO: piperonyl-butoxide
*Kdr*-w: West African *kdr*-allele (1014F)
*kdr*-e: East African *kdr* allele (1014S)
