# Supplementary figures and images for: A survey of Anopheles species composition and insecticide resistance on the island of Bubaque, Bijagos Archipelago, Guinea-Bissau
Source: Malar J. 2020 Jan 15;19:27. doi: 10.1186/s12936-020-3115-1 (PMC6964033; doi:10.1186/s12936-020-3115-1)

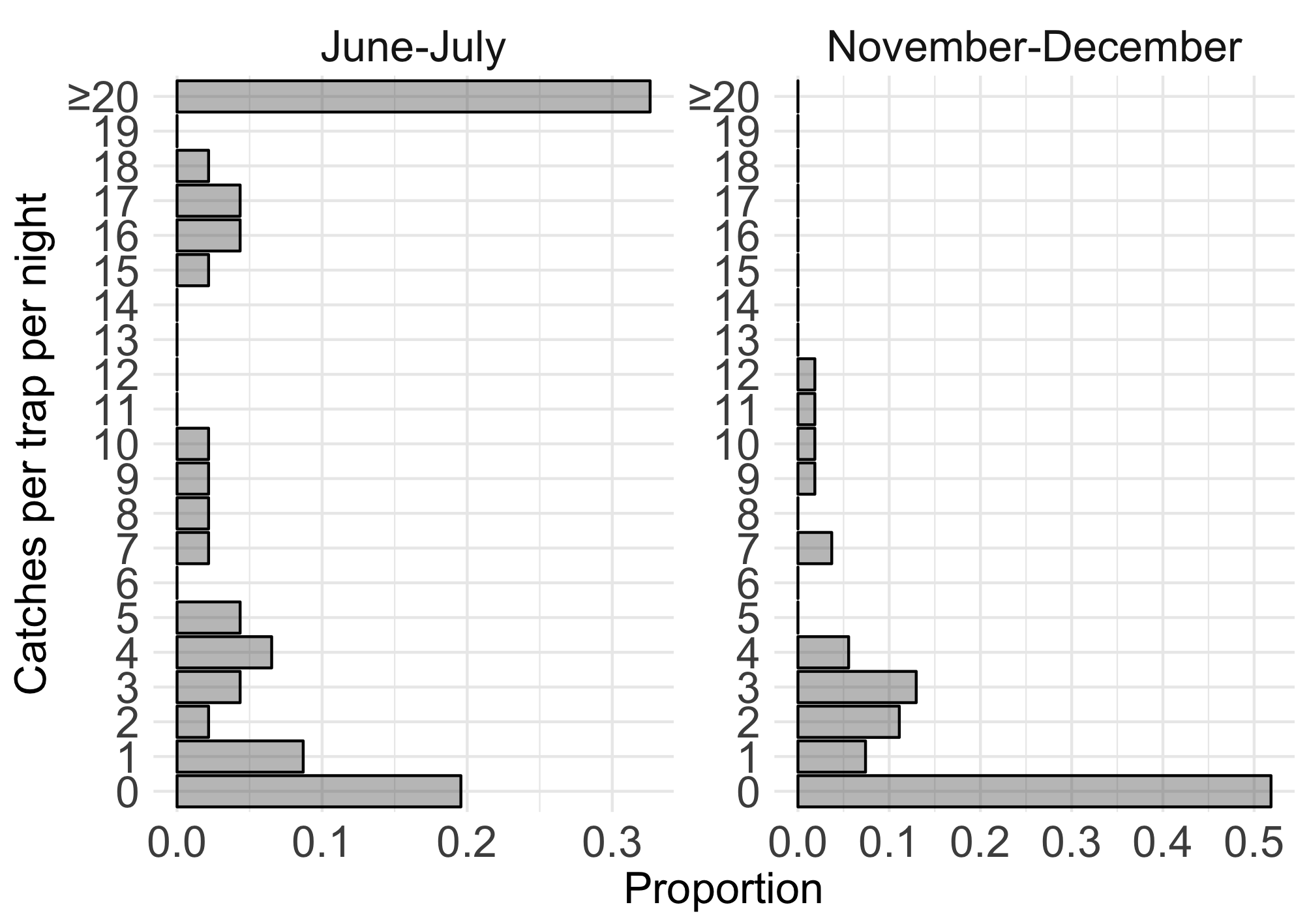

Supplement: Supplementary file 1 — Additional file 1: Figure S1. Number of female Anopheles mosquitoes caught per CDC light trap per night during the June-July and November–December trapping. [file 12936_2020_3115_MOESM1_ESM.tiff]
