# Supplementary material for: A survey of Anopheles species composition and insecticide resistance on the island of Bubaque, Bijagos Archipelago, Guinea-Bissau
Source: Malar J. 2020 Jan 15;19:27. doi: 10.1186/s12936-020-3115-1 (PMC6964033; doi:10.1186/s12936-020-3115-1)
Supplement: Supplementary file 2 — Additional file 2: Table S1. Larval sampling site description and species found. [file 12936_2020_3115_MOESM2_ESM.docx]

**Table S1.** Larval sampling site description and species found.

| **Species** | **Lat** | **Long** | **Site description** | **Identification** |
| --- | --- | --- | --- | --- |
| *Anopheles gambiae* | 11.30541 | -15.83684 | Urban | PCR |
| *Anopheles gambiae* | 11.29341 | -15.82882 | Urban | PCR |
| *Anopheles gambiae* | 11.29093 | -15.82952 | Urban | PCR |
| *Anopheles gambiae* | 11.29185 | -15.85906 | Agricultural | PCR |
| *Anopheles gambiae* | 11.29097 | -15.85634 | Agricultural | PCR |
| *Anopheles gambiae* | 11.21932 | -15.86943 | Non-urban | PCR |
| *Anopheles coluzzii* | 11.29747 | -15.84026 | Urban | PCR |
| *Anopheles coluzzii* | 11.29593 | -15.84574 | Non-urban | PCR |
| *Anopheles coluzzii* | 11.26706 | -15.86245 | Urban | PCR |
| *Anopheles coluzzii* | 11.23437 | -15.87824 | Village | PCR |
| *An. gamb*/*col hyb* | 11.30231 | -15.83497 | Urban | PCR |
| *An. gamb*/*col hyb* | 11.30067 | -15.83618 | Urban | PCR |
| *An. gamb*/*col hyb* | 11.29794 | -15.82791 | Urban | PCR |
| *An. gamb*/*col hyb* | 11.28510 | -15.83144 | Non-urban | PCR |
| *An. melas* | 11.30085 | -15.84704 | Mangrove | PCR |
| *An. melas* | 11.28838 | -15.82439 | Coastal | PCR |
| *An. melas* | 11.27791 | -15.83571 | Mangrove | PCR |
| *An. melas* | 11.27591 | -15.83441 | Coastal | PCR |
| *An. melas* | 11.26203 | -15.88185 | Coastal | PCR |
| *An. melas* | 11.23245 | -15.86640 | Coastal | PCR |
| *Culex rubinotus* | 11.29394 | -15.82823 | Urban | Morph |
| *Culex theileri* | 11.29409 | -15.82814 | Urban | Morph |
| *Aedes aegypti* | 11.30385 | -15.83621 | Urban | Morph |
| *Toxorhynchites* spp. | 11.30385 | -15.83621 | Urban | Morph |
